# Assessment of pathogenic variation in gynecologic cancer genes in a national cohort

**DOI:** 10.1038/s41598-023-32397-8

**Published:** 2023-03-31

**Authors:** Urška Kotnik, Aleš Maver, Borut Peterlin, Luca Lovrecic

**Affiliations:** 1 Clinical Institute of Genomic Medicine, University Medical Centre Ljubljana, Ljubljana, Slovenia; 2 Faculty of Medicine, University of Ljubljana, Ljubljana, Slovenia; 3 Biotechnical Faculty, University of Ljubljana, Ljubljana, Slovenia

**Keywords:** Cancer genetics, Gynaecological cancer, Genetic testing, Population screening

## Abstract

Population-based estimates of pathogenic variation burden in gynecologic cancer predisposition genes are a prerequisite for the development of effective precision public health strategies. This study aims to reveal the burden of pathogenic variants in a comprehensive set of clinically relevant breast, ovarian, and endometrial cancer genes in a large population-based study. We performed a rigorous manual classification procedure to identify pathogenic variants in a panel of 17 gynecologic cancer predisposition genes in a cohort of 7091 individuals, representing 0.35% of the general population. The population burden of pathogenic variants in hereditary gynecologic cancer-related genes in our study was 2.14%. Pathogenic variants in genes ATM, BRCA1, and CDH1 are significantly enriched and the burden of pathogenic variants in CHEK2 is decreased in our population compared to the control population. We have identified a high burden of pathogenic variants in several gynecologic cancer-related genes in the Slovenian population, most importantly in the BRCA1 gene.

## Introduction

Hereditary gynecologic cancers include breast, ovarian, and endometrial cancer, as well as some other rare cancers of the female reproductive system^1^, where a pathogenic variant, leading to a distinctly increased lifetime risk of cancer development, has been discovered in a cancer-related gene^2^. Altogether, pathogenic variants in gynecologic cancer predisposition genes account for 5–10% of breast, ovarian, and endometrial cancer^3,4^ with carriers of pathogenic variants in these genes having a significantly increased lifetime risk for developing cancer (up to 72%)^5^. However, the burden of hereditary gynecologic cancer has not been established in the general population^6^, since the disease is characterized by both late-onset and incomplete penetrance^7^.

Furthermore, genomic data varies between populations, even between different European populations^17^. For instance, it has been shown that genetic testing in Slovenian patients with breast and ovarian cancer consistently yields very high BRCA1 and BRCA2 detection rates compared to patients from other populations^8^. It is imperative that population-specific studies are conducted to gain insight into the genetic diversity of pathogenic findings in specific populations.

Public health genomics is a field, concerned with the assessment of the health characteristics of a particular population by evaluating the impact of genetic risk factors on the population’s health status as well as disease burden^9–11^. By determining the genetic risk factors burden, such as pathogenic variation in genes in a population-based study, we are able to evaluate the likelihood of pathogenic findings in the general population^12^, which can be used to develop precision medicine-based population health strategies, such as population-specific disease prevention, screening, and surveillance^10^. Hereditary cancers, specifically hereditary gynecologic cancers, have been noted as a priority in public health genomics^11^.

A few studies focused on the burden of pathogenic and likely pathogenic variants in genes associated with different cancer syndromes in unaffected individuals in population-based cohorts, namely analyzing large international control databases^13,14^, population-based biobanks^15^, or individuals who underwent exome sequencing for other conditions^16,17^. The burden of pathogenic variation in unaffected individuals has been determined in the control cancer-free population in case–control studies as well^6,18–20^. A wide prevalence range of pathogenic variants in unaffected individuals was reported in these studies (from 0.5 to 4.8%), depending on the methodology and the number of genes analyzed^17,20^. Yet, only a few previous studies have examined the population burden of gynecologic cancer predisposition genes beyond BRCA1/2 and Lynch syndrome genes in national cohorts^18,19,21^.

Several genes have previously been associated with gynecologic cancer development^7^ and even more are currently being evaluated as candidate gynecologic cancer predisposition genes^22^. Professional organizations have published screening and/or treatment guidelines for carriers of pathogenic variants in only a limited number of genes, with strong evidence for cancer predisposition^7^, making them clinically significant, as they present actionable information to patients. Discoveries in the dynamic field of cancer research are published daily and thus making a panel of clinically significant genes a moving target; however, leading professional organizations have published lists of genes that they deem clinically significant^7,23,24^. There is a lack of large-scale population-based studies providing assessments of pathogenic variants burden in a comprehensive panel of clinically significant breast, ovarian and endometrial cancer predisposition genes in the general population^6^.

To develop precision population health strategies, this study aims to assess the population burden of germline pathogenic variants in clinically actionable gynecologic cancer predisposition genes in the Slovenian general population, consequently estimating the burden of individuals with hereditary cancer syndromes on a population level.

## Methods

### Ethical approval

All experimental protocols were approved by the Medical Ethics Committee of the Republic of Slovenia (0120-170/2022/03).

### Study population

We investigated cancer variants in exome datasets of 7091 individuals who were referred to the Clinical Institute of Genomic Medicine, University Medical Centre, Ljubljana, Slovenia from July 2014 to May 2022 for exome sequencing for various rare genetic conditions other than cancer. This data is stored in the Slovenian genomic database. All patients gave informed consent for participation and all methods were performed in accordance with the Declaration of Helsinki. The patient data were non-reciprocally de-identified identified and aggregated data on variant frequencies was assessed. GnomAD non-cancer population was chosen as a control for our study (N = 134,187).

### Sequencing and variant calling

The samples for exome sequencing were enriched using TruSight One, TruSight Exome, and Nextera Coding Exome capture kits by Illumina or Agilent SureSelect Human All Exon v2 and Agilent SureSelect Human All Exon v5 capture kits by Agilent Technologies. Sequencing was performed on Illumina MiSeq or Illumina HiSeq 2500 platforms. A minimum median exome coverage was 60x, with over 95% of targets covered with at least 10 × sequencing depth. Raw sequence files were processed using a custom exome analysis pipeline and aligned to UCSC hg19 human reference genome as previously described by our group^25^.

### Gene panel

We constructed an innovative gene panel containing genes associated with gynecologic cancer. Several professional resources were examined for gynecologic cancer-related genes: NCCN guidelines for hereditary breast and ovarian cancer^7^, Genomics England PanelApp panels for gynecologic cancers^23^, and ClinGen’s expert panel recommendations^24^. Only genes with definite associations and available clinical recommendations were included in our gene panel: PanelApp’s green list (15 genes), NCCN’s strong evidence level (16 genes), and definite association by ClinGen’s expert panels (17 genes). Genes defined as having a strong connection by multiple of those sources were included in our panel. We were able to construct an innovative gene panel encompassing 17 genes causative of breast, ovarian, and/or endometrial cancer (Table 1). The presence of pathogenic variants in this panel of 17 gynecologic cancer-related genes has not been analyzed in an unaffected population before.

**Table 1:** Genes and associated cancer phenotypes.

Genes	Transcript	Gene MIM number	Phenotype and gynecologic cancer susceptibility	Disorder MIM number
ATM	NM_000051.4	607585	Breast cancer susceptibility	114480
BARD1	NM_000465.4	601593	Breast cancer susceptibility	114480
BRCA1	NM_007294.4	113705	Hereditary breast and ovarian cancer	604370
BRCA2	NM_000059.4	600185	Hereditary breast and ovarian cancer	612555
BRIP1	NM_032043.3	605882	Breast and ovarian cancer susceptibility	114480
CDH1	NM_004360.5	192090	Breast cancer susceptibility	114480
CHEK2	NM_007194.4	604373	Breast cancer susceptibility	114480
MLH1	NM_000249.4	120436	Lynch syndrome (Endometrial and ovarian cancer susceptibility)	609310
MSH2	NM_000251.3	609309	Lynch syndrome (Endometrial and ovarian cancer susceptibility)	120435
MSH6	NM_000179.3	600678	Lynch syndrome (Endometrial and ovarian cancer susceptibility)	614350
PALB2	NM_024675.4	610355	Breast cancer susceptibility	114480
PMS2	NM_000535.7	600259	Lynch syndrome (Endometrial and ovarian cancer susceptibility)	614337
PTEN	NM_001304717.5	601728	Cowden syndrome (Breast and endometrial cancer susceptibility)	158350
RAD51C	NM_058216.3	602774	Breast and ovarian cancer susceptibility	613399
RAD51D	NM_002878.4	602954	Breast and ovarian cancer susceptibility	614291
STK11	NM_000455.5	602216	Peutz-Jeghers syndrome (Breast, ovarian, and endometrial cancer susceptibility)	175200
TP53	NM_000546.5	191170	Li-Fraumeni syndrome (Breast cancer susceptibility)	151623

### Variant filtering

The variant filtering process was based on both effect (functional effect, clinical impact) and frequency of the variants in control populations, where found variants had to satisfy both types of criteria to be included.

Firstly, the functional effect was assessed. The criterion for inclusion was either for the variant to have a moderate or high impact on protein function (inframe insertion/deletion, frameshift, missense, canonical splice site variant, stop/start loss, stop gain, UTR region deletion) or to have a high effect on protein splicing (> 0.5 SNV rf or ada score)^26^. Secondly, the variant’s frequency in control populations (gnomAD and our whole dataset) was evaluated. Variants were excluded from further evaluation for appearing in > 10 heterozygotes in the gnomAD population or > 20 individuals in our dataset and for being present in more than three individuals in the homozygous state in gnomAD or our dataset.

The remaining variants were filtered based on their ClinVar classification. Variants classified as benign and likely benign or conflicting between benign/likely benign and a variant of uncertain significance in the ClinVar database were excluded from further analysis. Furthermore, variants in noncoding regions and synonymous variants not predicted to affect splicing that were not present in ClinVar or were classified as VUS in ClinVar were excluded as well.

The only exception was known pathogenic variants (as evaluated by multiple unanimous submissions in ClinVar database^27^), where the effect and frequency filtering criteria were not enforced.

All remaining variants were evaluated using the ACMG guidelines^28^ and classified as a pathogenic, likely pathogenic, variant of uncertain significance, likely benign, or benign.

### Overview of variant classification

ACMG criteria^28^ were applied to the filtered variants for annotation and classification of genetic variants based on population frequency, functional effects of variants, published research, and more. We have considered population frequency, as it is recorded in GnomAD v.2.1.1^29^ (for criteria PS4, PM2_SUP, BA1, BS1) (accessed 5th of May 2022). PM2 was used as a supporting criterion PM2_SUP^30^. The benign frequency cut-off was adopted from Varsome calculations^31^ (BS1) (accessed 14th of March 2022). The loss of function variants were classified according to ClinGen’s algorithm^32^. The PVS1 criterion was considered for loss of function variants, appearing in genes, fulfilling ClinGen’s criteria for haploinsufficiency. Null variants in coding exons before the last 50 nucleotides of the penultimate exon and variants where known pathogenic variants were appearing downstream of the said variant, were predicted to undergo nonsense-mediated decay. A variant was considered to affect a biologically relevant transcript if it was present in an exon with a pext score of > 0.5 (sourced from gnomAD). In variants, appearing in canonical splice sites, disruption of the reading frame was assessed. Z-scores were collected from gnomAD and missense variants in genes, for which z-score exceeded 3.09^33^ were assigned PP2 criteria. ClinVar^27^ was used for the identification of known pathogenic variants (for criteria PS1 and PM5). We have used an agreement of multiple predictive in silico tools as a measurement of the pathogenicity of the variants (PP3, BP4). Conservation was assessed using PhastCons^34^ and splice sites were identified based on Human Splicing Finder 3.1^35^ (BP7). Prevalence of variants in cases vs controls and previous records in patients (PS4), functional studies (PS3, BS3), and previous accounts of variant segregation (PP1, BS4) were extracted from the literature. PS4 criterion was used according to ClinGen’s guidelines^36^. PS4_STR was assigned to variants, appearing in > 0.001% of the gnomAD population, for which case–control studies reported OR > 5. Extremely rare variants (< 0.001% of the gnomAD population) were divided into three categories: PS4_STR, PS4_MOD, and PS4_SUP, according to the number of patients with variant previously reported: > 4 STR, > 2 MOD, > 1SUP^36^. Data regarding repeat regions were extracted from RepeatMasker via UCSC Genome Browser^37^ (PM4, BP3). Data on functional domains were extracted from UniProt^38^ (PM1). Criteria PP5 and BP6 were not used because of recent recommendations against their use^39^. This work was performed on data without identifiers or phenotypes, so genotype–phenotype correlations were not possible and criteria PS2, PM3, PM6, PP4, BP2, and BP5 were not applied. Specific classification criteria by ClinGen were used to classify variants in the ATM gene^40^. For the PMS2 gene, only variants, appearing in the non-homologous regions of the PMS2 gene, as assessed by Alignability of 100mers by GEM (via UCSC Browser) were considered. Rules for combining criteria for the classification of variants were adopted from ACMG guidelines^28^ with amendments by classification modeling guidelines by Tavtigian et al.^41^. Most importantly, variants with a very strong and a moderate criterion were classified as pathogenic and variants with two strong criteria were classified as likely pathogenic. Variants with PVS1 and PM2_SUP criteria only were classified as likely pathogenic according to ClinGen’s recommendation^30^.

### Statistical analysis

The differences between the burden of pathogenic variants in the study and control populations were calculated by Chi-squared test (χ2 test), with two-tailed analysis. We considered p < 0.05 as statistically significant. Odds ratios (OR) at the 95% confidence interval for each gene were calculated as well.

### Ethics declaration

All patients gave informed consent for participation and all methods were performed in accordance with the Declaration of Helsinki. All data was de-identified.

## Results

### Variant filtering and classification

**Figure 1:**
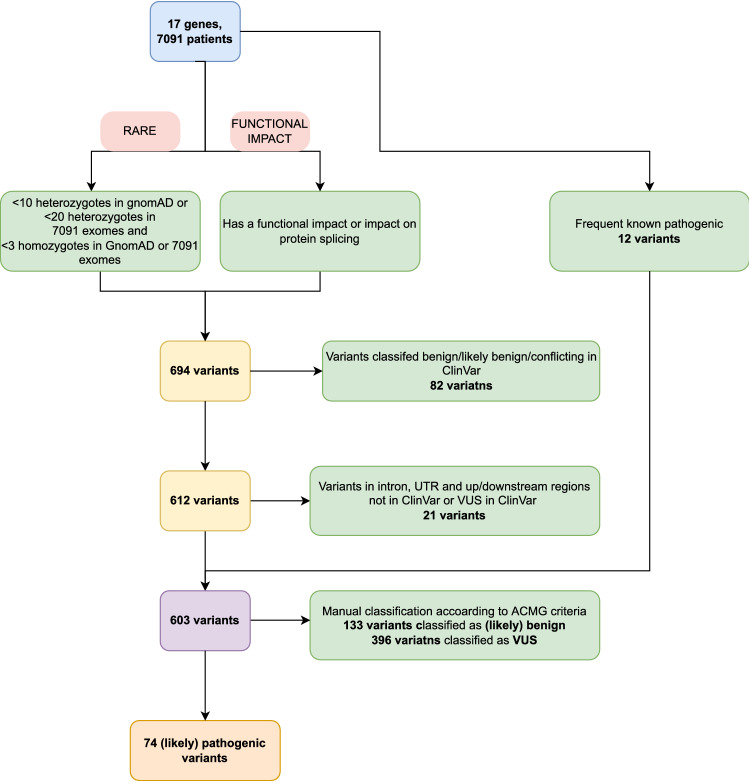
Variant filtering and classification process. Amidst 694 rare and functional or known pathogenic variants found in our population, 107 variants were classified as benign (B), likely benign (LB), or a variant of uncertain significance (VUS) during the filtering process (filtered variants). Firstly, 82 variants were classified as B, LB, or conflicting between B, BL, and VUS based on their ClinVar classification. Next, variants in noncoding regions and synonymous variants that were not present in ClinVar or were classified as VUS in ClinVar were assigned VUS status (21 variants). We added 12 variants to the classification group since they are known pathogenic variants, frequent in control populations and cancer patients. The resulting 603 variants were classified manually according to the American College of Medical Genetics and Genomics and the Association for Molecular Pathology (ACMG) standards and guidelines for the interpretation of sequence variants (ACMG guidelines). We classified 603 variants into five categories: pathogenic (P), likely pathogenic (LP), variant of uncertain significance, and likely benign or benign. Six variants were classified as benign, 127 were likely benign, 396 were VUS, of which ten were conflicting between B/LB and P/LP and there was not enough data to classify the remaining 381 variants into either B/LB or P/LP categories. Lastly, 14 variants were likely pathogenic and 60 were classified as pathogenic.

Altogether, 649 rare and functional variants in 17 genes were found in our study population of 7091 exomes. After the variant filtering process resulted in 603 variants, manual classification was employed and 74 unique pathogenic and likely pathogenic variants in 13 cancer genes were found, appearing one or more times in the Slovenian genomic database (Fig. 1). No pathogenic/likely pathogenic variants were found in four of the investigated genes.

### The burden of (likely) pathogenic variants in our study population and control population

The burden of likely pathogenic and pathogenic variants in our cohort was 2.14%, 152 heterozygotes were found to carry a pathogenic or likely pathogenic variant in a gene linked to gynecologic cancer. The most frequently found variants were in BRCA1 and BRCA2 genes, present in 0.40% and 0.25% of the studied population, respectively. Heterozygous (likely) pathogenic variants in ATM were present in 0.51% of our population. CHEK2 had a variant prevalence of 0.31%. Variants in mismatch repair (MMR) genes, associated with Lynch syndrome (MLH1, MSH2, MSH6, and PMS2 genes) were present in 20 heterozygotes (10 in PMS2) and altogether appeared in 0.28% of the studied population. Pathogenic variants in RAD51C and PALB2 were present in 0.13% of our cohort, pathogenic variants in BARD1 were present in 0.06% of heterozygotes and pathogenic variants in BRIP1 and CDH1 had a prevalence of 0.04% (Table 2).

**Table 2:** Pathogenic and likely pathogenic variants in the Slovenian population in 17 gynecologic cancer genes.

Genes	Number of P/LP variants	Number of heterozygotes with P/LP variants	Frequency of heterozygotes with P/LP variants in Slovenian population (%) (N = 7091)	Frequency of heterozygotes with P/LP variants in controls (%) (N = 134.187)	Odds ratio (OR) (95% CI)	Chi-square statistic (two-tailed)	Chi-square p-value
ATM	18	36	0.51	0.29	**1.73 CI 1.23–2.43**	10.171	**0.00143**
BARD1	4	4	0.06	0.06	0.96 CI 0.35–2.62	0.007	0.934
BRCA1	10	28	0.40	0.22	**1.81 CI 1.22–2.66**	9.150	**0.00249**
BRCA2	6	18	0.25	0.30	0.84 CI 0.52–1.34	0.549	0.459
BRIP1	3	3	0.04	0.09	0.45 CI 0.14–1.40	2.007	0.157
CDH1	3	3	0.04	0.01	**6.31 CI 1.71–23.31**	10.050	**0.00152**
CHEK2	8	22	0.31	0.57	**0.54 CI 0.36–0.83**	8.136	**0.00434**
MLH1	3	3	0.04	0.03	1.67 CI 0.51–5.44	0.741	0.389
MSH2	2	2	0.03	0.01	2.37 CI 0.54–10.29	1.401	0.236
MSH6	5	5	0.07	0.09	0.80 CI 0.33–1.96	0.235	0.628
PALB2	5	9	0.13	0.15	0.87 CI 0.45–1.70	0.158	0.691
PMS2	4	10	0.14	0.12	1.18 CI 0.62–2.24	0.266	0.606
PTEN	NA	NA	NA	0.01	NA	NA	NA
RAD51C	3	9	0.13	0.09	1.47 CI 0.74–2.89	1.248	0.264
RAD51D	NA	NA	NA	0.04	NA	NA	NA
STK11	NA	NA	NA	NA	NA	NA	NA
TP53	NA	NA	NA	0.02	NA	NA	NA
Sum	74	152	2.14	2.10	1.02 CI 0.87–1.21	0.073	0.787

*P/LP* pathogenic/likely pathogenic, *OR* odds ratio, *CI* confidence interval.

Statistically significant differences are in bold.

Chi-squared test with two-tailed analysis was used and odds ratios were calculated to enable a comparison of Slovenian population frequencies of pathogenic variants to control the population of the gnomAD database (Table 2). The Slovenian population was statistically significantly (p < 0.05) enriched for pathogenic variants in ATM, BRCA1, and CDH1 genes when compared to the gnomAD non-cancer control population. Pathogenic variants in CHEK2 had a lower prevalence in the Slovenian population when compared to the control population, mainly due to the low prevalence of the c.1100del variant in the Slovenian population; its prevalence is five times higher in the control population (0.04% vs 0.21%). A statistically significant difference could not be established for the other genes from our study.

### Description of likely pathogenic and pathogenic variants in our study

We have identified 74 distinct pathogenic and likely pathogenic variants in our study population in the following 13 cancer-related genes: ATM, BARD1, BRCA1, BRCA2, BRIP1, CDH1, CHEK2, MLH1, MSH2, MSH6, PALB2, PMS2, in RAD51C (Supplementary Table S1).

Pathogenic and likely pathogenic variants in BRCA1 and BRCA2 genes were present in 0.65% of our population. The most common pathogenic variants in BRCA genes in our cohort were c.5266dupC, c.181T>G, and c. 1687C>T in the BRCA1 gene and c.3975_3978dupTGCT and c.7806-2A>G in the BRCA2 gene, which have been previously reported to be the five most common pathogenic variants in BRCA1/2 genes in Slovenian HBOC families, representing 67% of pathogenic variants in Slovenian cancer patients^42^; together, they represent 71% of the pathogenic variation in BRCA genes in our study. The most frequent variant in our population is c.5266dupC in BRCA1, which is the one most common pathogenic BRCA1 variants worldwide^43^. The most common variant in BRCA2 is c.7806-2A>G, a Slovenian founder variant^44^. Two BRCA1 variants, previously not described in the Slovenian population, are c.3331_3334delCAAG and c.4065_4068delTCAA, which have been identified in other populations in breast and ovarian cancer patients^45,46^. Two BRCA2 variants, 1813delA, and c.8755-1G>A have not been described in the Slovenian population yet, but have been previously described in other populations^47,48^.

ATM represents the most frequently mutated gene in our population (likely pathogenic/ pathogenic variants were present in 0.51% of the cohort). Most of the variants are known pathogenic variants, however, three of the found variants (c.2007T>A, c.7452_7453delAT, and c.8708delC) were previously unreported in scientific literature and were classified as likely pathogenic based on their loss of function effect (frameshift and stop gained) and absence in the control population of gnomAD project. Variants in MMR genes (MLH1, MSH2, MSH6, and PMS2) were found in 20 heterozygotes and together appeared in 0.28% of the studied population. These variants are known pathogenic variants, described in many populations worldwide in patients with Lynch-related cancers^49–51^. Eight different pathogenic and likely pathogenic variants were found in the CHEK2 gene in our study, representing 0.31% of our cohort. This is statistically significantly lower than the prevalence in the control database (p < 0.05). Three of the found variants, c.444+1G>A, c.1100del, and c.349A>G, were previously described in the Slovenian population amongst the most frequent CHEK2 pathogenic variants in Slovenian CHEK2 positive breast cancer patients^52^. Pathogenic variants in RAD51C and PALB2 were present in 0.13% of our cohort. Pathogenic variants in CDH1 had a prevalence of 0.04% in our cohort, a substantial enrichment in comparison to the gnomAD population (p < 0.05). The cumulative prevalence of pathogenic variants in BARD1 was 0.06% and in the BRIP1 gene it was 0.04%; among those, BARD1 variants c.1381G>T and c.1538T>G, and BRIP1 variants c.318delT and c.368C>A, had never been described in scientific literature or databases; yet their loss of function effect and absence from control populations led to the classification as (likely) pathogenic. No pathogenic and likely pathogenic variants were detected in our study population for RAD51D, STK11, PTEN, or TP53.

## Discussion

The population burden of pathogenic variants in hereditary gynecologic cancer-related genes in Slovenia is 2.14%, according to our study. The estimation is based on an analysis of pathogenic variants in 17 clinically significant gynecologic cancer predisposition genes, in a population-based cohort of 7091 unaffected individuals, representing 0.35% of the Slovenian population. Further analysis revealed that our population is enriched for pathogenic variants in ATM, BRCA1, and CDH1 genes and that prevalence of pathogenic variants in the CHEK2 gene is lower in the Slovenian population, when compared to GnomAD non-cancer control population. This estimate presents the first report of the burden of population burden of pathogenic variants associated with any disease in Slovenia one of the first to show the burden of gynecologic cancer-related pathogenic variants on a population level and as such warrants both scientific and clinical attention.

In the Slovenian general population, the highest burden of (likely) pathogenic variants was found in the BRCA1 and BRCA2 genes, 0.65%, with 0.40% in BRCA1 and 0.25% in BRCA2. Until recently, the prevalence of pathogenic variants in BRCA genes in the general population has been estimated to be between 1:300 and 1:500 (0.2–0.3%)^53^. However, based on population studies, this fact has recently been challenged. An early study on exomes of 60.706 individuals from the ExAC project has shown that the burden of pathogenic variants BRCA genes is closer to 1:161 (0.62%)^14^, and several recent studies have reported higher than before thought burden of pathogenic variants in the different cancer-free cohorts^15,54,55^. Our results support the claim that the burden of BRCA pathogenic variants in the unaffected population might be higher than traditionally estimated. Furthermore, we report two unique features of the Slovenian population: a high prevalence of pathogenic variants in the BRCA1 gene compared to the control population, which is reflected in high detection rates of pathogenic variants in BRCA genes in Slovenian HBOC patients as well^8^ and a higher burden of pathogenic BRCA1 variants compared to BRCA2 pathogenic variants in the Slovenian population. In most populations, the BRCA2 pathogenic variants prevail^6^, however, a higher burden of BRCA1 pathogenic variants has been previously described in cancer patient populations from a few eastern and central European countries^56^.

A high burden of several other cancer-predisposing genes was reported in our study. Pathogenic or likely pathogenic variants in the ATM gene are the most frequent (0.51%) and compared to that of gnomAD, the prevalence of ATM (likely) pathogenic variants in the Slovenian population is significantly increased (p = 0.00143). Pathogenic variants in MMR genes (MLH1, MSH2, MSH6, and PMS2), that are involved in the development of Lynch syndrome are present in 0.28% of our study population. Compared to other populations (0.18% in a recent large study in the Chinese population^57^), the percentage is high and is close to that of BRCA1/2, and ATM pathogenic variants. The distribution of pathogenic variants among MMR genes in our study seems remarkable: 50% of variants were found in PMS2, 25% in MSH6, and 15% in MLH1 and 10% in MSH2, as it is not reflective of the distribution of pathogenic variants in MMR genes in cancer populations, where up to 90% of variants are found in MLH1 and MSH2 genes^58^. The high burden of PMS2 variants may be explained by some recent findings and general facts: firstly, PMS2 pathogenic variants have low penetrance as discovered in a recent study^59^; secondly, a significant part of the pathogenic variation in MMR genes is due to large-scale deletions^60^, and this variation, not included in our analysis, might contribute to the redistribution of pathogenicity among the MMR genes; and finally, Lynch syndrome is grossly underdiagnosed^61^, leaving MSH2 and PMS2 pathogenic variant carriers frequently unidentified^62^, therefore, current studies of pathogenic variants distribution in recognized Lynch syndrome patients may not reflect the true distribution either in the patient group or in the general population. The burden of pathogenic variants in the CHEK2 gene is statistically significantly decreased in the Slovenian population compared to the gnomAD population (p = 0.0043), mostly due to the low prevalence of the c.1100del variant in our population. This variant is very common in Northern and Western European populations^63^ and these populations are the main component of the gnomAD cohort^29^, explaining the high prevalence in the gnomAD population. Moreover, this variant has been rarely found in Southern European and non-European populations^64,65^. Our study supports the claim, that the prevalence of this variant is less frequent outside of the Northern European populations. Seven previously unreported likely pathogenic variants in four gynecologic cancer genes (ATM, BARD1, and BRIP1) were found in the Slovenian genomic database^40^. In light of the fact that these variants were discovered in our apparently non-cancer-affected population, additional functional studies will be required to demonstrate their pathogenic effect. We have not discovered any pathogenic variants in RAD51D, STK11, PTEN, and TP53 genes in our non-cancer affected study population of 7091 participants; this is not surprising since most of these genes had none to a very low pathogenic variant prevalence in control populations in previous studies^66^.

While several studies were undertaken to reveal the carrier rate of cancer pathogenic variants in unaffected populations^6,15,16^, ours is the first study to evaluate this particular panel of clinically significant gynecologic cancer-related genes in a general population, therefore, making the comparability of the burden in our population (2.14%) challenging. A recent study in a Swiss population has analyzed the prevalence of pathogenic variants in a panel of breast and ovarian cancer genes in a non-cancer cohort^16^. The study enrolled only 400 non-cancer cases undergoing exome sequencing and included 19 genes, having 16 genes coinciding with our study. The prevalence of pathogenic variants in this unaffected population (2.2%) is similar to our study. Another study included a large sample of 32.544 unaffected women as controls and reported the prevalence of pathogenic variants in 12 breast-cancer-related genes (11 the same as in our study) to be 1.63%^6^, which is a reasonably similar result considering the lower number of genes included. The differences between studies may be partially explained by differences in the methodological approach, most importantly in gene selection and classification methodology, different sample sizes of the previous studies, as well as unique population characteristics such as the presence of Slovenian founder BRCA2 variant in our study^44^ and high burden of BRCA1 pathogenic variants in the Slovenian population^8^.

Our work is notable for the large cohort size, a manual review of variants using the ACMG standards for interpretation^28^, and an exclusive focus on the gynecologic cancer genes. There are, however, some limitations to our study design. The anonymization of our study disabled the use of patient phenotypes and family history in the classification of the variants. As a result, it is possible that some of the patients who underwent exome sequencing at our institution for non-cancer Mendelian disorders may harbor a personal or family history of cancer, in addition to the primary reason for their genetic analysis. Family history of cancer in carriers and potential additional segregation analysis might allow some variants to be upgraded. Secondly, our analysis does not include copy number variation analysis or methylation analysis, as some of this variation may be significant in cancer predisposition risk, therefore, prevalence calculations might be slightly underestimated. Nevertheless, even without considering this additional variation, an important number of unaffected individuals in our study have been found to be carriers of a pathogenic variant in a gynecologic cancer predisposition gene.

This study provides an assessment of the burden of pathogenic variation in a panel of clinically significant hereditary gynecologic cancer genes in a general population. Understanding the population burden of hereditary gynecologic cancer will ultimately lead to the development and implementation of precision public health strategies in a sector of hereditary gynecologic cancer and is expected to help bridge the gap between an individual and the population’s health in public health genomics. Our dataset of unaffected individuals has a high number of carriers of pathogenic variants of several clinically actionable genes, especially BRCA1, and this presents an opportunity to discuss population or opportunistic genomic screening^67^, a strategy that has been often suggested as a cost-effective tool to discover pre-symptomatic carriers^68^.

In conclusion, we report a population burden of 2.14% of the pathogenic variation in clinically actionable gynecologic cancer-related genes in a large non-cancer population in a Slovenian population-based study.

## Supplementary Information

Supplementary Information.

## Data Availability

The datasets generated and/or analyzed during the current study are available in the ClinVar repository (accession numbers SCV002762771 to SCV002762841).
