# Supplementary material for: Assessment of pathogenic variation in gynecologic cancer genes in a national cohort
Source: Sci Rep. 2023 Mar 31;13:5307. doi: 10.1038/s41598-023-32397-8 (PMC10066348; doi:10.1038/s41598-023-32397-8)
Supplement: Supplementary file 1 — Supplementary Information. [file 41598_2023_32397_MOESM1_ESM.docx]

**Supplementary table 1: Pathogenic and likely pathogenic variants**

| **Gene** | **Nomenclature** | **Variant type** | **Nucleotide change** | **Amino acid change** | **Number of heterozygotes** | **ACMG criteria** | **Classification** |
| --- | --- | --- | --- | --- | --- | --- | --- |
| ATM | NM_000051.4 | Frameshift | c.1333del | p.Gln445fs | 1 | PVS1, PM2_SUP, PM3_SUP | Pathogenic |
| ATM | NM_000051.4 | Frameshift | c.1564_1565del | p.Glu522fs | 9 | PVS1, PM3_VSTR | Pathogenic |
| ATM | NM_000051.4 | Stop gain | c.2007T>A | p.Cys669* | 1 | PVS1, PM2_SUP | Likely pathogenic (N) |
| ATM | NM_000051.4 | Stop gain | c.2341C>T | p.Gln781* | 1 | PVS1, PM2_SUP, PM3_SUP | Pathogenic |
| ATM | NM_000051.4 | Synonymous | c.3576G>A | p.Lys1192Lys | 2 | PS3_MOD, PM3_VSTR | Pathogenic |
| ATM | NM_000051.4 | Stop gain | c.3802del | p.Val1268* | 2 | PVS1, PS3_MOD, PM3_VSTR | Pathogenic |
| ATM | NM_000051.4 | Stop gain | c.5515C>T | p.Gln1839* | 1 | PVS1, PM2_SUP, PM3_SUP | Pathogenic |
| ATM | NM_000051.4 | Stop gain | c.5932G>T | p.Glu1978* | 2 | PVS1, PS3_MOD, PM3_VSTR | Pathogenic |
| ATM | NM_000051.4 | Frameshift | c.6082del | p.Gln2028fs | 1 | PVS1, PM2_SUP, PM3_SUP | Pathogenic |
| ATM | NM_000051.4 | Missense | c.6095G>A | p.Arg2032Lys | 3 | PS3_MOD, PM3_VSTR | Pathogenic |
| ATM | NM_000051.4 | Stop gain | c.67C>T | p.Arg23* | 1 | PVS1, PM3_VSTR | Pathogenic |
| ATM | NM_000051.4 | Frameshift | c.689del | p.Asn230fs | 4 | PVS1, PM2_SUP, PM3_SUP | Pathogenic |
| ATM | NM_000051.4 | Stop gain | c.7096G>T | p.Glu2366* | 1 | PVS1, PM2_SUP, PM3_VSTR | Pathogenic |
| ATM | NM_000051.4 | Frameshift | c.7452_7453del | p.Phe2485fs | 1 | PVS1, PM2_SUP | Likely pathogenic (N) |
| ATM | NM_000051.4 | Canonical splice | c.7630-2A>C |  | 1 | PVS1, PS3_MOD, PM2_SUP, PM3_VSTR | Pathogenic |
| ATM | NM_000051.4 | Missense | c.8147T>C | p.Val2716Ala | 2 | PS3_MOD, PM3_VSTR, PM5 | Pathogenic |
| ATM | NM_000051.4 | Stop gain | c.8425C>T | p.Gln2809* | 2 | PVS1, PM2_SUP, PM3_SUP | Pathogenic |
| ATM | NM_000051.4 | Frameshift | c.8708del | p.Pro2903fs | 1 | PVS1, PM2_SUP | Likely pathogenic (N) |
| BARD1 | NM_000465.4 | Canonical splice | c.216-1G>T |  | 1 | PVS1, PS4_SUP, PM2_SUP | Pathogenic |
| BARD1 | NM_000465.4 | Stop gain | c.1381G>T | p.Gly461* | 1 | PVS1, PM2_SUP | Likely pathogenic (N) |
| BARD1 | NM_000465.4 | Stop gain | c.1538T>G | p.Leu513* | 1 | PVS1, PM2_SUP | Likely pathogenic (N) |
| BARD1 | NM_000465.4 | Stop gain | c.1690C>T | p.Gln564* | 1 | PVS1, PS3, PS4_STR | Pathogenic |
| BRCA1 | NM_007294.4 | Missense | c.116G>A | p.Cys39Tyr | 1 | PS3, PS4_STR, PM2_SUP, PM5 | Pathogenic |
| BRCA1 | NM_007294.4 | Stop gain | c.1687C>T | p.Gln563* | 5 | PVS1, PS3, PS4_STR | Pathogenic |
| BRCA1 | NM_007294.4 | Missense | c.181T>G | p.Cys61Gly | 7 | PS3, PS4_STR, PM5_STR, PP1 | Pathogenic |
| BRCA1 | NM_007294.4 | Frameshift | c.843_846del | p.Ser282fs | 1 | PVS1, PS4_STR, PM2_SUP | Pathogenic |
| BRCA1 | NM_007294.4 | Frameshift | c.844_850dup | p.Gln284fs | 1 | PVS1, PS4_STR, PM2_SUP | Pathogenic |
| BRCA1 | NM_007294.4 | Frameshift | c.2269del | p.Val757fs | 1 | PVS1, PS4_STR PM2_SUP | Pathogenic |
| BRCA1 | NM_007294.4 | Frameshift | c.3018_3021del | p.His1006fs | 1 | PVS1, PS4_STR PM2_SUP | Pathogenic |
| BRCA1 | NM_007294.4 | Frameshift | c.3331_3334del | p.Gln1111fs | 1 | PVS1, PS3, PS4_STR PM2_SUP | Pathogenic |
| BRCA1 | NM_007294.4 | Frameshift | c.4065_4068del | p.Asn1355fs | 1 | PVS1, PS4_STR | Pathogenic |
| BRCA1 | NM_007294.4 | Frameshift | c.5266dup | p.Gln1777fs | 9 | PVS1, PS4_STR, BS1 | Pathogenic |
| BRCA2 | NM_000059.4 | Frameshift | c.1813del | p.Ile605fs | 1 | PVS1, PS4_STR | Pathogenic |
| BRCA2 | NM_000059.4 | Frameshift | c.3975_3978dup | p.Ala1327fs | 5 | PVS1, PS4_STR PM2_SUP | Pathogenic |
| BRCA2 | NM_000059.4 | Frameshift | c.5351dup | p.Asn1784fs | 1 | PVS1, PS4_STR PM2_SUP | Pathogenic |
| BRCA2 | NM_000059.4 | Canonical splice | c.7806-2A>G |  | 7 | PVS1, PS3, PS4_STR, PM2_SUP | Pathogenic |
| BRCA2 | NM_000059.4 | Missense | c.8351G>A | p.Arg2784Gln | 3 | PS3, PS4_STR | Likely pathogenic |
| BRCA2 | NM_000059.4 | Canonical splice | c.8755-1G>A |  | 1 | PVS1, PS3, PS4_STR, PM2_SUP, PP1 | Pathogenic |
| BRIP1 | NM_032043.3 | Frameshift | c.318del | p.His107fs | 1 | PVS1, PM2_SUP | Likely pathogenic (N) |
| BRIP1 | NM_032043.3 | Stop gain | c.368C>A | p.Ser123* | 1 | PVS1, PM2_SUP | Likely pathogenic (N) |
| BRIP1 | NM_032043.3 | Frameshift | c.2684_2687del | p.Ser895fs | 1 | PVS1, PS4_STR | Pathogenic |
| CDH1 | NM_004360.5 | Stop gain | c.583C>T | p.Gln195* | 1 | PVS1, PS4_STR, PM2_SUP | Pathogenic |
| CDH1 | NM_004360.5 | Synonymous | c.1137G>A | p.Thr379Thr | 1 | PS4_STR, PS3, PM2_SUP, PP1 | Pathogenic |
| CDH1 | NM_004360.5 | Canonical splice | c.1565+1G>A |  | 1 | PVS1, PS4_STR, PS3, PM2_SUP, PP1 | Pathogenic |
| CHEK2 | NM_007194.4 | Stop gain | c.85C>T | p.Gln29* | 2 | PVS1, PS4_SUP, PM2_SUP | Pathogenic |
| CHEK2 | NM_007194.4 | Missense | c.349A>G | p.Arg117Gly | 6 | PS3, PS4_STR | Likely pathogenic |
| CHEK2 | NM_007194.4 | Missense | c.433C>T | p.Arg145Trp | 1 | PS3, PS4_STR, PP1 | Likely pathogenic |
| CHEK2 | NM_007194.4 | Canonical splice | c.444+1G>A |  | 7 | PVS1, PS3, PS4_STR, BS1 | Pathogenic |
| CHEK2 | NM_007194.4 | Canonical splice | c.683+2T>C |  | 1 | PVS1, PM2_SUP, PS4_SUP | Pathogenic |
| CHEK2 | NM_007194.4 | Frameshift | c.1100del | p.Thr367fs | 3 | PVS1, PS3, PS4_STR, BS1 | Pathogenic |
| CHEK2 | NM_007194.4 | Missense | c.1169A>C | p.Tyr390Ser | 1 | PS3, PS4_STR | Likely pathogenic |
| CHEK2 | NM_007194.4 | Frameshift | c.1263del | p.Ser422fs | 1 | PVS1, PS4_STR | Pathogenic |
| MLH1 | NM_000249.4 | Missense | c.244A>G | p.Thr82Ala | 1 | PS3, PS4_STR, PM2_SUP, PM5, PP1 | Pathogenic |
| MLH1 | NM_000249.4 | Canonical splice | c.1039-1G>T |  | 1 | PVS1, PS4_SUP, PM2_SUP | Pathogenic |
| MLH1 | NM_000249.4 | Missense | c.1896G>C | p.Glu632Asp | 1 | PS4_STR, PM2_SUP, PM5, PP1, PP3 | Likely pathogenic |
| MSH2 | NM_000251.3 | Stop gain | c.952G>T | p.Glu318* | 1 | PVS1, PS4_SUP, PM2_SUP | Pathogenic |
| MSH2 | NM_000251.3 | Stop gain | c.1165C>T | p.Arg389* | 1 | PVS1, PS4_STR, PM2_SUP | Pathogenic |
| MSH6 | NM_000179.3 | Missense | c.2314C>T | p.Arg772Trp | 1 | PS4_STR, PS3, PM2_SUP, PM5, PP1 | Pathogenic |
| MSH6 | NM_000179.3 | Missense | c.2906A>G | p.Tyr969Cys | 1 | PS4_STR, PM5, PP1 | Likely pathogenic |
| MSH6 | NM_000179.3 | Frameshift | c.3261dup | p.Phe1088fs | 1 | PVS1, PS4_STR, BS1 | Pathogenic |
| MSH6 | NM_000179.3 | Stop gain | c.3539C>G | p.Ser1180* | 1 | PVS1, PS4_SUP, PM2_SUP | Pathogenic |
| MSH6 | NM_000179.3 | Canonical splice | c.3647-1G>A |  | 1 | PVS1, PS4_MOD, PM2_SUP, PP1 | Pathogenic |
| PALB2 | NM_024675.4 | Frameshift | c.172_175del | p.Gln60fs | 1 | PVS1, PS4_STR | Pathogenic |
| PALB2 | NM_024675.4 | Frameshift | c.509_510del | p.Arg170fs | 4 | PVS1, PS3, PS4_STR | Pathogenic |
| PALB2 | NM_024675.4 | Stop gain | c.1027C>T | p.Gln343* | 1 | PVS1, PS4_STR, PM2_SUP, PP1 | Pathogenic |
| PALB2 | NM_024675.4 | Stop gain | c.1451T>A | p.Leu484* | 2 | PVS1, PS4_STR, PM2_SUP | Pathogenic |
| PALB2 | NM_024675.4 | Stop gain | c.2192T>G | p.Leu731* | 1 | PVS1, PS4_SUP, PM2_SUP | Pathogenic |
| PMS2 | NM_000535.7 | Frameshift | c.211_214del | p.Asn71fs | 2 | PVS1, PM2_SUP, PS4_SUP | Pathogenic |
| PMS2 | NM_000535.7 | Stop gain | c.400C>T | p.Arg134* | 4 | PVS1, PS3, PS4_STR, PM2_SUP | Pathogenic |
| PMS2 | NM_000535.7 | Synonymous | c.825A>G | p.Gln275Gln | 3 | PS3, PS4_SUP, PM2_SUP | Likely pathogenic |
| PMS2 | NM_000535.7 | Canonical splice | c.2007-2A>G |  | 1 | PVS1, PS4_STR, PM2_SUP | Pathogenic |
| RAD51C | NM_058216.3 | Missense | c.404G>A | p.Cys135Tyr | 1 | PS3, PS4_STR, PM2_SUP, PM5 | Pathogenic |
| RAD51C | NM_058216.3 | Canonical splice | c.572-1G>C |  | 6 | PVS1, PS4_SUP, PM2_SUP | Pathogenic |
| RAD51C | NM_058216.3 | Canonical splice | c.706-2A>G |  | 2 | PVS1, PS3, PS4_STR, PP1 | Pathogenic |

*Legend: N= novel variants in the Slovenian population*
